# Editorial: Aerobic and anaerobic fermentation of gaseous and liquid one carbon feedstocks to produce food, feed, biopolymers and value-added products

**DOI:** 10.3389/fbioe.2024.1334864

**Published:** 2024-02-06

**Authors:** Maximilian Lackner, Ildar Mustakhimov, Juan B. García Martínez, Stefan Pflügl

**Affiliations:** ^1^ Circe Biotechnologie GmbH, Vienna, Austria; ^2^ Technische Universität Wien, Institute of Chemical, Environmental and Bioscience Engineering, Vienna, Austria; ^3^ Institute of Biochemistry and Physiology of Microorganisms (RAS), Pushchino, Russia; ^4^ Alliance to Feed the Earth in Disasters, Fairbanks, United States

**Keywords:** C1, gas fermentation, syngas, biogas, bioplastics, biopolymers, single cell protein, aerobic and anaerobic processes

Dear readers, We are proud to present you 9 articles by 54 leading authors from industry and academia around the Research Topic of microbial utilization of one-carbon feedstocks and gas fermentation. Fermentation is among the oldest technologies of mankind, and biocatalysts are known to offer low-cost and selective production of various target products, from commodities such as fuels to high-value compounds such as bioactive and pharmaceutical ingredients. First generation biofuels have shown that competition over agricultural land can lead to detrimental effects, amongst them price pressure on feed and food, direct and indirect land use change, putting pressure on our climate, and resource consumption, e.g., of fertilizers and water. Biobased and/or biodegradable polymers (bioplastics), alternative proteins, and other compounds of interest should ideally be made from waste resources, such as agricultural and forest residues, or industrial waste streams (both liquid and gas). Significant research efforts have been undertaken to develop more efficient processes for lignocellulosic hydrolysis, to provide second generation fermentable sugars, but it has yet to fulfill scaling projections. An alternative concept is to convert waste biomass into gaseous feedstocks, amongst them biogas (from wet biomass) and synthesis gas (from dry biomass), yielding well-defined feedstocks in the form of CO, CO_2_/H_2_ and CH_4_, which can be subjected to gas fermentation. Alternatively, (electro)chemical catalysis can be used to generate syngas, methanol or formate from CO_2_ and renewable electricity or H_2_. CO_2_ is in this context can either originate from industrial point sources or direct air capture. Methanol and CH_4_ have already been used at scale, and with renewable electricity becoming more widely available, power-to-gas processes and electrosynthesis open the way for additional one-carbon feedstocks such as CO_2_ which is also available from various point sources.

This Research Topic includes two articles describing the potential of aerobic methane utilization and upgrading into single cell protein (García Martínez and coworkers) or PHB (Safaeian and coworkers). Salusjärvi and coworkers describe a potential bioproduction scenario for the biopolymer precursors beta-alanine and L-lactic acid using metabolic engineering of the aerobic, facultative lithoautotrophic strain *Rhodococcus opacus*. Production of both compounds could be demonstrated using CO_2_, H_2_ and O_2_, and in the case of beta-alanine also in an electrobioreactor set-up using CO_2_ and providing H_2_ and O_2_ by water electrolysis. Another litoautotroph, the well-studied gram-negative *Cupriavidus necator* was adapted by Wickham-Smith and coworkers to higher tolerance of carbon monoxide, thus enabling the use of syngas by *C. neactor* in bioproduction processes. The work of Dahlin and coworkers is another example of one carbon utilization with a non-model organism, in this case the microalgae *Picochlorum renovo* which was engineered for photoformatotrophy, i.e., utilization of formate driven by energy harvested from light. Shirvani and coworkers presented a concept for a process chain from biomass to methanol using biomass gasification and chemical syngas-to-methanol conversion to provide the model feedstock. It could be demonstrated that the model aerobic, methylotrophic yeast *Komagataella phaffii* is able to grow on ammonia generated during biomass gasification as a by-product, thus paving the way for nitrogen recovery from low-value biogenic feedstocks. In the context of methylotrophy, Singh and coworkers review the development of microbial platforms to enable a methanol-based bioindustry. Anaerobic one-carbon feedstock utilization by acetogens is another interesting route for carbon- and energy-efficient bioproduction. The work of Nogle and coworkers in this Research Topic describes the discovery of a plasmid replication origin in the model acetogen *Clostridium autoethanogenum* and its utilization in heterologous expression of the pathway for isopropanol production. Finally, a thermostable and anaerobic fluorescent marker is described by Hocq and coworkers which was used to characterize a promoter library in the thermophilic acetogen *Thermoanaerobacter kivui*.

